# Dual Temperature and Metal Salts-Responsive Interpenetrating Polymer Networks Composed of Poly (*N*-isopropylacrylamide) and Polyethylene Glycol

**DOI:** 10.3390/polym13111750

**Published:** 2021-05-27

**Authors:** Junta Sano, Shigeki Habaue

**Affiliations:** Department of Applied Chemistry, College of Engineering, Chubu University, 1200 Matsumoto-cho, Kasugai, Aichi 487-8501, Japan; tk19801-6197@sti.chubu.ac.jp

**Keywords:** poly(*N*-isopropylacrylamide), polyethylene glycol, interpenetrating polymer networks, hydrogel, metal salt sensing

## Abstract

Novel interpenetrating polymer networks (IPNs) composed of poly(*N*-isopropylacrylamide) (poly-NIPAM) and polyethers—namely, polyethylene glycol (PEG) and poly(tetramethylene oxide)—were synthesized in the absence and presence of polysiloxane containing a silanol residue. Gelation was accomplished using end-capped polyethers with trimethoxysilyl moieties and proceeded through simultaneous radical gelation of NIPAM and condensation of the silyl groups to form siloxane linkages. Thus, a novel one-step method constructing an IPN structure was provided. The obtained IPNs showed a gentle temperature-responsive volume change in water owing to the constructed poly-NIPAM gel component. In addition, a specific color-change response to chemical stimuli, such as CuCl_2_ and AgNO_3_ in water, was observed only when both components of poly-NIPAM and PEG existed in a gel form. For example, a single network gel composed of poly-NIPAM or PEG was isolated as a pale blue hydrogel, whereas IPNs composed of poly-NIPAM and PEG components turned yellow after swelling in an aqueous CuCl_2_ solution (0.1 M, pale blue). Dual-responsive functionalities of the synthesized hydrogels to temperature and metal salts, along with volume and color changes, were demonstrated.

## 1. Introduction

Hydrogels are hydrophilic polymer networks that hold large amounts of aqueous media. They have been frequently applied in biomedical fields, drug delivery, separation, self-healing technologies, etc.; thus, development of hydrogels is an area with a high potential. For example, chitosan hydrogels for ocular application and use as a 3D printing material were recently reported [1,2,3]. A hydrogel that changes (typically in volume) when exposed to external stimuli, such as temperature, pH, chemicals, and electricity, is called an intelligent or smart hydrogel [4,5,6]. A typical smart hydrogel is the poly(*N*-isopropylacrylamide) (poly-NIPAM) gel, which undergoes a volume phase transition at a low critical solution temperature of approximately 32 °C. Poly-NIPAM can be combined with other polymers, such as poly(acrylic acid) and polyethylene glycol (PEG), which contribute biocompatibility, permeability, metal complex formation ability, and hydrophilicity to form a gel with superior properties and functions. For instance, NIPAM is often combined with telechelic-type PEG macromonomers bearing vinyl groups and macroinitiators, forming gels with block and branched copolymer structures [7,8,9,10].

Interpenetrating polymer network (IPN) gels, consisting of two or more networks that are physically entangled but not covalently bonded, are attractive new materials because they synergistically affect the properties of the network and improve the gel strength. Various applications, including recent developments such as a nano-filtration membrane, have been made [11,12,13]. Therefore, the IPN is a valuable option for smart hydrogels [14,15,16]; furthermore, it can be applied to dual-responsive materials, which respond to two factors. Among such smart IPN hydrogels, temperature- and pH-responsive hydrogels have been extensively studied because these two factors can be easily controlled in an aqueous phase [4,17,18,19,20]. Thus, the smart IPNs that multiply responding to the various external stimuli in aqueous media are still limited. The preparation methods of IPNs can be divided into two categories: sequential and simultaneous. In sequentially formed IPNs, one network is swollen in a solution of precursors of the other. The precursors are then formed into their own network. In simultaneous formation, the respective precursors of both networks are left to react at the same time. The latter method is more restricted because two reactions constructing respective networks must proceed without interfering with each other, but it represents a simple and useful process. Development of a novel one-step simultaneous method is, therefore, valuable. Thus far, IPN gels with a PEG network have been synthesized stepwise from macromonomers, such as diacrylate PEG [21,22].

Recently, we demonstrated a novel simultaneous method that produces IPN gels composed of polysiloxane and poly(acrylamide) networks through radical gelation of acrylamides in the presence of a crosslinking agent and siloxane Si–O bond formation from silanol (–SiOH) groups on the polysiloxane [23,24]. The resulting organic–inorganic hybrid IPN gels are easily formed with no interference between the radical addition to the vinyl group and the inorganic silanol-condensation reaction. In this procedure, the polysiloxanes containing the silanol residue are simply prepared by a partial silylation reaction between the silanols on polymeric silicic acid (poly-SOL) derived from silica gel [25,26,27,28] and various trialkoxysilane-type silylation reagents in tetrahydrofuran (THF). The polysiloxane used here can be characterized by simple functionalization through this process, in marked contrast to the conventional poly(dialkylsiloxane).

PEG hydrogel can be also prepared by inorganic-type condensation of PEG end-capped with trialkoxysily [–Si(OR)_3_] groups, and has a structure significantly different from those conventionally formed from PEG macromonomers with vinyl groups and macroinitiators [29,30]. The trialkoxysily end-caps are easily introduced by reacting PEG with two equivalents of (3-isocyanatopropyl)trialkoxysilane, leading to a urethane linkage. That is, a one-step simultaneous method can synthesize novel IPN gels composed of PEG and poly(acrylamide), such as poly-NIPAM, from a mixture of silylated PEG, NIPAM, and a radically crosslinking agent. In addition, the polysiloxane-containing silanol residue co-exists during the reactions, enabling the construction of a double network-type structure through silylation between the reactive silyl group attached on PEG and the silanols.

**Scheme 1:**
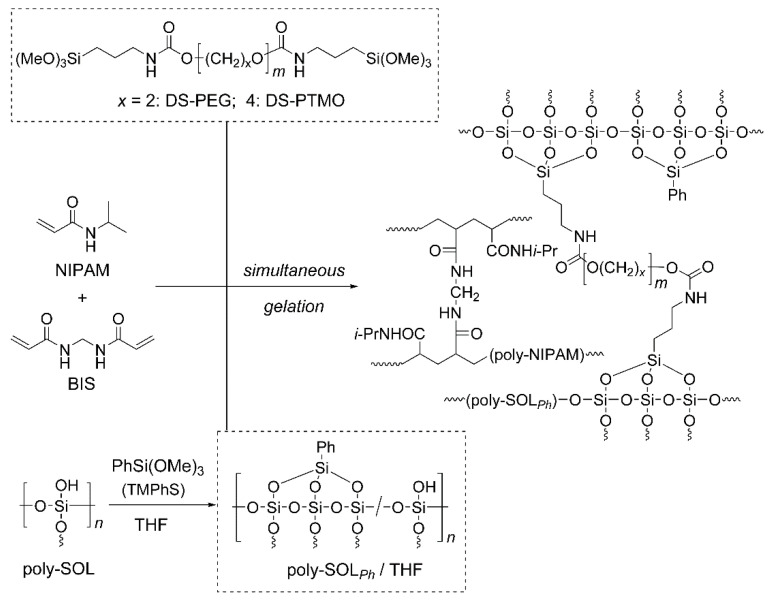
IPN synthesis with disilylated polyethers and NIPAM in the presence of polysiloxane-containing silanol groups.

In the present study, end-capped polyethers [PEG and poly(tetramethylene oxide) (PTMO) with telechelic trimethoxysily moieties, DS-PEG and DS-PTMO, respectively] were prepared and reacted with NIPAM, a cross-linker [*N,N’*-methylenebisacrylamide (BIS)], and a radical initiator [azobisisobutyronitrile (AIBN)], in the presence or absence of polysiloxane-containing silanol residue. After the reaction, IPN gels were formed. The polysiloxane (poly-SOL_Ph_) was synthesized from poly-SOL with a 0.2 equivalent of phenyltrimethoxysilane (PhTMS) to the used silica [27] (Scheme 1). Thus, a novel simultaneous gelation was successfully attained. The obtained hydrogels changed their volume in response to a temperature change. The temperature-responsive behavior was enabled by the poly-NIPAM network, while the polyether structure of the other network could coordinately bind metal salts. The response of the hydrogels to various metal salts as external chemical stimuli was also examined to develop a novel dual responsive system. The IPN gels with the PEG chain showed characteristic behaviors along with a color change in response to aqueous CuCl_2_ and AgNO_3_ solutions.

## 2. Materials and Methods

### 2.1. Measurements

Fourier transform infrared (FT-IR) spectra were recorded using a Shimadzu IR Affinity-1S spectrometer (Shimadzu, Kyoto, Japan). Scanning electron micrographs (SEM) were captured using a JEOL JMS-6510LA (JEOL, Akishima, Japan). The gel strengths (jelly strengths) of the obtained hydrogels were tested with a Sun rheometer CR-100 (Sun Scientific, Tokyo, Japan) (test speed, 60 mm/min; stroke displacement, 4 or 5 mm; plunger diameter, 5 mm ϕ; temperature, 25 °C).

### 2.2. Materials

The silica gel [spherical (particles of size 63–210 μm), neutral] (Kanto, Tokyo, Japan), silylation reagents [(3-isocyanatopropyl)trimethoxysilane (purity, >97%; IPTMS) and PhTMS (>98%)], polyethers [PEG with molecular weight (M_w_) = 2000 and PTMO with M_w_ = 2000], and the catalyst [dibutyltin dilaurate (DBTD), extra pure grade (TCI, Tokyo, Japan)] were used as received. The vinyl monomer NIPAM and cross-linker BIS (Wako, Osaka, Japan) were purified by recrystallization before use. The IPN gel was synthesized in THF solvent with a radical initiator, azobisisobutyronitrile (>98%, AIBN) (Wako) and was chemically stimulated by various metal salts [purities >99%; (CH_3_CO_2_)K, >97%; CuCl_2_, >95% (practical grade)] and a 0.1 M aqueous solution of AgNO_3_ (*f* = 1.000, Wako).

### 2.3. Preparation of Disilylated Polyethers

The disilylated polyethers DS-PEG and DS-PTMO were generated in situ by reacting their corresponding polyethers with IPTMS in the presence of DBTDL (molar ratio 1:2:0.01) in THF (35 wt.%) for 2 h at 50 °C, then for 1 h at room temperature under a N_2_ atmosphere. The prepared THF solutions were used without isolation.

A solution of DS-PEG or DS-PTMO in THF (1.5 mL) was introduced to a perfluoroalkoxyl (PFA) resin test tube and was left standing still for 20 h at 60 °C to allow the gel formation [29]. The obtained product was dried overnight under reduced pressure at 80 °C. The homogeneous gels prepared from DS-PEG and DS-PTMO, called G**_PEG_** and G**_PTMO_**, respectively, were isolated with almost quantitative yields of 0.480 and 0.517 g, respectively.

**Figure 1:**
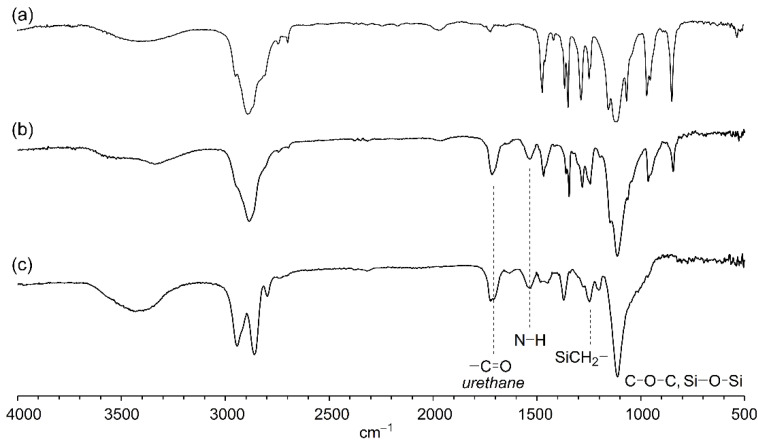
FT-IR spectra of (**a**) PEG and gels, (**b**) G**_PEG_** prepared from DS-PEG and (**c**) G**_PTMO_** prepared from DS-PTMO (KBr).

Figure 1 presents the FT-IR spectra of the obtained gels and the starting material (PEG). The characteristic absorptions of the C=O (1712 cm^−1^) and N–H (1529 cm^−1^) bonds in the urethane linkages and silyl groups, such as Si–C (1250 cm^−1^) and Si–O–Si (1110 cm^−1^), along with those of the polyether unit, were clearly observed. These results indicated that the polyether gels were effectively produced by the condensation reaction of the end-capped silyl groups of the polyethers.

### 2.4. IPN Synthesis

A THF solution of poly-SOL_Ph_ was prepared from poly-SOL (2.0 mL, 1.6 M, theoretical) with PhTMS (0.2 equivalent to the used silica). This solution was used without isolation, as previously reported [27].

NIPAM (0.90 g, 8.0 mmol), BIS, and AIBN ([NIPAM]/[BIS]/[AIBN] = 100/4/1) were introduced to the poly-SOL_Ph_ (2 mL) solution in a PFA test tube. In the reaction without poly-SOL_Ph_, the polysiloxane solution was replaced with THF (2 mL). After adding the DS-PEG or DS-PTMO solution (1.5 mL), the mixture was gelated for 24 h at 60 °C under a N_2_ atmosphere. The obtained gel was immersed first in THF for 2 days, then in distilled water for 2 days. Finally, it was dried overnight under reduced pressure at 80 °C.

The swelling degrees of the obtained gels were evaluated by the conventional gravimetric method [23]. The gels were immersed in an excess amount of distilled water or an aqueous metal–salt solution for 24 h at an appropriate temperature. When swollen, the gels were removed from the medium and weighed. The degree of swelling (*Q*) was calculated as

*Q* = (*m*_w_ − *m*_d_)/*m*_d_ (1)

where *m*_d_ and *m*_w_ are the weights of the dried and wet samples, respectively.

## 3. Results and Discussion

### 3.1. Synthesis of IPNs

**Figure 2:**
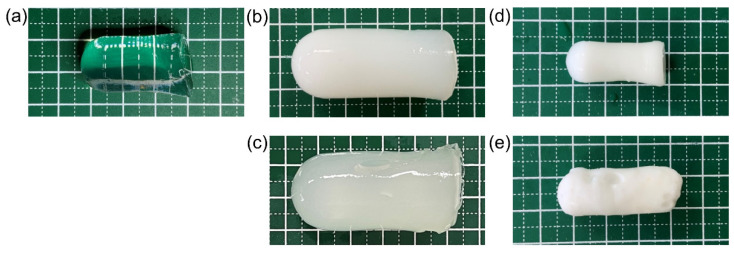
Photographs of the obtained hydrogels: (**a**) poly-NIPAM gel (G_NIPAM_), (**b**) IPN_PEG_ (Table 1, run 1), (**c**) IPN_SOL_ *_+_*_PEG_ (run 2), (**d**) IPN_PTMO_ (run 3), and (**e**) IPN_SOL_ *_+_*_PTMO_ (run 4).

Table 1 lists the yields and swelling degrees of the IPNs synthesized from the end-capped polyethers, NIPAM, and BIS in the presence and absence of the reactive polysiloxane, poly-SOL_Ph_. A single NIPAM network, G_NIPAM_, was prepared under the same conditions (NIPAM = 0.90 g, [NIPAM]/[BIS]/[AIBN] = 100/4/1; THF = 3.5 mL; 96% yield). The gelation was effective and afforded opaque white hydrogels after washing in excess water; in contrast, the G_NIPAM_ hydrogel was clear, as shown in Figure 2. After drying, hard gels were isolated in good yield. For example, gelation with DS-PEG and no poly-SOL**_Ph_** produced 1.12 g of a THF- and water-insoluble product, IPN_PEG_ (run 1). Although the poly-SOL_Ph_ addition slightly reduced the yield of the gel product (runs 2 and 4), the method easily introduced a functional group into the PEG network. In this instance, a phenyl moiety was attached to the gel as described later.

**Figure 3:**
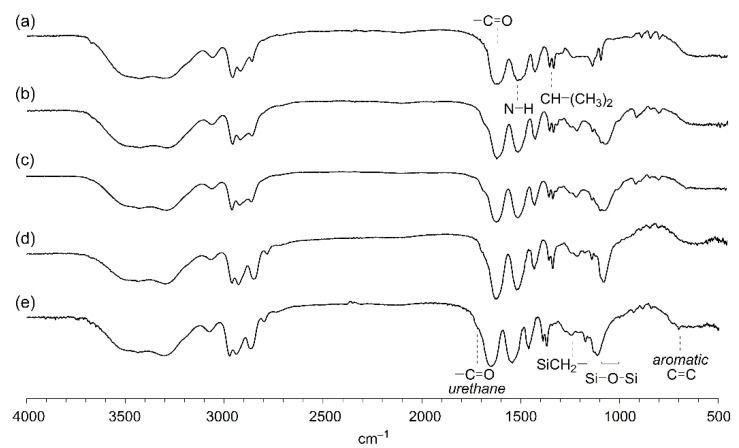
FT-IR spectra of (**a**) poly-NIPAM gel (**G*_NIPAM_***), (**b**) IPN_PE_***_G_*** (Table 1, run 1), (**c**) IPN_SOL + PEG_ (run 2), (**d**) IPN_PTMO_ (run 3), and (**e**) IPN_SOL + PTMO_ (run 4) (KBr).

Figure 3 presents the FT-IR spectra of the obtained gels (Table 1, runs 1–4) and G_NIPAM_. The spectra display the characteristic absorptions of the C=O bond (1700 cm^−1^) in the urethane linkages and the C–O–C group (1100 cm^−1^) of the polyether unit, along with the absorptions of poly-NIPAM (C=O, 1640 cm^−1^; N–H, 1540 cm^−1^; C–(CH_3_)_2_, 1386 and 1367 cm^−1^). The spectra of the IPN_SOL *+* PEG_ and IPN_SOL *+* PTMO_ gels additionally exhibited the characteristic bending-vibration band of the aromatic group around 690 cm^−1^, indicating that these gels contained the poly-SOL_Ph_ component. These results indicated that the IPN structure was successfully constructed during gelation.

The IPN system can improve the poor mechanical properties of the poly-NIPAM gel [14,15,16]. The strengths of the G_NIPAM_, IPN_PTMO_ (Table 1, run 3), and IPN_SOL + PTMO_ (run 4) hydrogels were simply estimated using a rheometer equipped with a 5 mm ϕ cylindrical plunger, which was depressed to a depth of 4 or 5 mm into the sample. The maximum test force was then detected. Here the hydrogel samples were cut so that their height almost equaled the stroke displacement. The strengths of the G_NIPAM_, IPN_PTMO_, and IPN_SOL + PTMO_ hydrogels were 0.035, 0.29, and 0.32 MPa, respectively. Note that the strength was nearly ten times higher in the IPN gels than in the poly-NIPAM gel, again supporting the IPN-type structure of the constructed gels.

**Figure 4:**
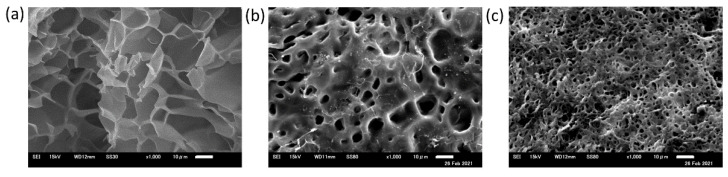
SEM images of fractured surfaces of the lyophilized gels: (**a**) poly-NIPAM gel (G_NIPAM_), (**b**) IPN_PEG_ (Table 1, run 1), and (**c**) IPN_SOL + PEG_ (run 2) (magnification, × 1000; scale bar, 10 μm). The gels were prepared by freeze-drying from water.

Figure 4 shows SEM images of the IPN_PEG_ and IPN_SOL + PEG_ cryogels isolated by freeze-drying from water. Images of G_NIPAM_ are shown for comparison. The gels exhibited a homogeneous sponge-like porous structure, but the morphologies greatly differed between G_NIPAM_ and the IPNs containing the PEG network. In addition, the pore size was much smaller in the IPN_SOL + PEG_ containing the polysiloxane component than in the IPN_PEG_ gel. According to these observations, the polymer components forming the IPN structures significantly affected the gel morphology.

### 3.2. Swelling Properties of the Obtained IPNs in Water

The swelling degrees *Q* of the IPNs were measured at 3 °C in water and are listed in Table 1. The *Q* value of the G_NIPAM_ hydrogel (13.8 at 3 °C) was much higher than those of the IPNs. The IPN_PEG_ and IPN_SOL + PEG_ gels containing a PEG chain tended to exhibit higher *Q*s than the IPN_PTMO_ and IPN_SOL + PTMO_ gels with a PTMO chain. Therefore, the hydrophilicity of the polyether chain influences the swelling properties of the synthesized IPNs.

**Figure 5:**
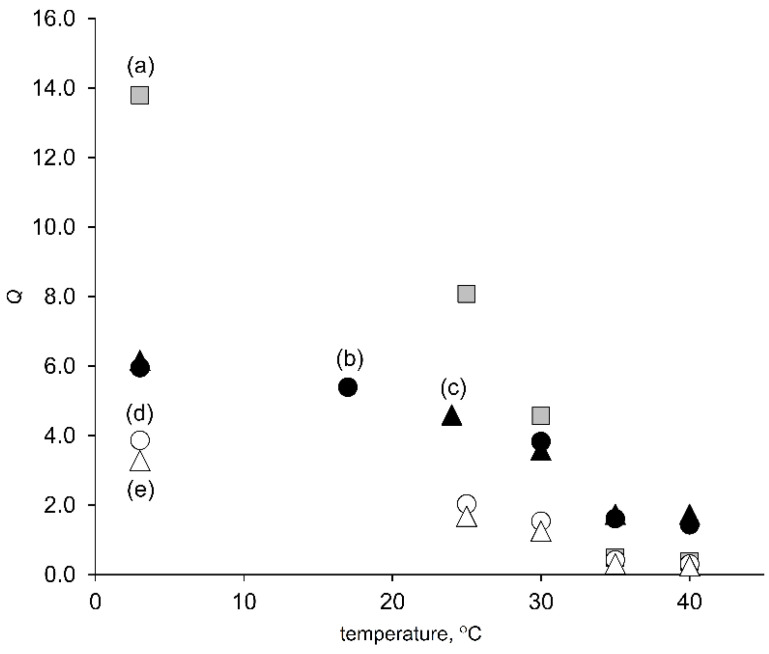
Temperature dependencies of swelling degrees (*Q*) of (**a**) the poly-NIPAM gel (G_NIPAM_) (■), (**b**) IPN_PEG_ (●, Table 1, run 1), (**c**) IPN_SOL + PEG_ (▲, run 2), (**d**) IPN_PTMO_ (○, run 3), and (**e**) IPN_SOL + PTMO_ (Δ, run 4) in water.

The temperature dependences of the swelling behaviors are depicted in Figure 5. The *Q* of the *G*_NIPAM_ hydrogel sharply decreased with temperature. The synthesized IPNs also responded to temperature with a volume change, but their *Q* values decreased more gently with increasing temperature. At temperatures above the volume phase transition temperature (VPTT) of the poly-NIPAM gel, the IPNs containing a PTMO chain demonstrated low *Q* values similar to that of G_NIPAM_, whereas the IPN_PEG_ and IPN_SOL + PEG_ gels demonstrated higher swelling behavior than G_NIPAM_. These results again suggested that the hydrophilic property of the polyether chain significantly affected the swelling behavior of the IPNs.

### 3.3. Responses of the Obtained Hydrogels to Metal–Salt Stimuli in Water

**Figure 6:**
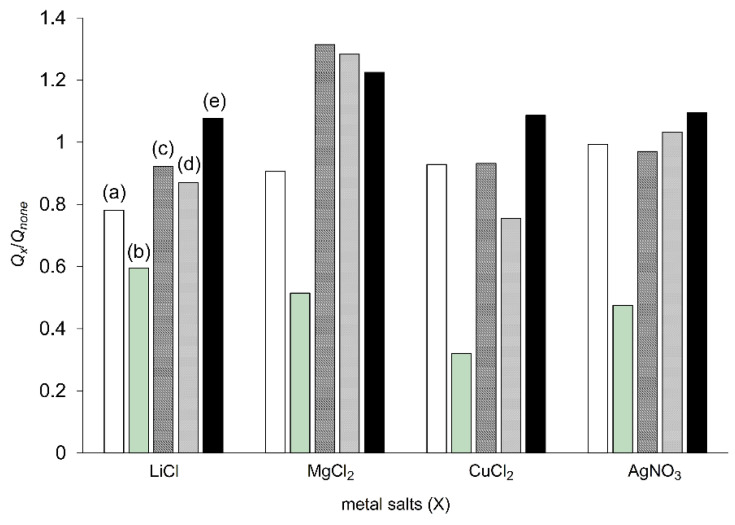
Swelling degree ratios (*Q*_X_/*Q*_none_) of (**a**, white bars) poly-NIPAM gel (G_NIPAM_), (**b**) IPN_SOL_ composed of poly-NIPAM and poly-SOL*_Ph_* [24], (**c**) IPN_PEG_ (Table 1, run 1), (**d**) IPN_SOL + PEG_ (run 2), and (**e**, black bars) IPN_SOL + PTMO_ (run 4) in the presence of metal salts (0.1 M) in water (24 °C).

The swelling responses of the gels in the presence of various metal salts (0.1 M) were tested in water at 24 °C. The observed behaviors were evaluated by their relative swelling ratios *Q*_X_/*Q*_none_, where *Q*_X_ and *Q*_none_ are the swelling degrees in the presence and absence of a metal salt (X), respectively. The IPN hydrogels were stable under the conditions of swelling experiments, and selected results are shown in Figure 6. The IPN_SOL_ gel, composed of poly-NIPAM and poly-SOL_Ph_ with no polyether component, was prepared as previously reported [24] for comparison.

The relative swelling ratio of the poly-NIPAM gel was less than 1.0, indicating that it shrunk during exposure to aqueous solutions of various metal salts (LiCl, MgCl_2_, CuCl_2_, and AgNO_3_). The estimated Q_X_/Q_none_ values of the IPN_SOL_ hydrogel were much lower than those of G_NIPAM_, indicating significant shrinkage in the presence of metal salts. This volume response was probably elicited by the hydrophobic property of the polysiloxane component. In contrast, when the IPN hydrogels containing polyether components (PEG and PTMO) were chemically stimulated by MgCl_2_, their Q_X_/Q_none_ values notably increased. This behavior might be attributed to the coordinately incorporated magnesium ions in the polyether comonents, which improved the swelling ability. Aqueous media containing typical metal salts (NaCl, CH_3_CO_2_K, (CH_3_CO_2_)_2_Ca, BaCl_2_, and LiCl), scarcely changed the volume of the IPNs. Therefore, the synthesized IPNs selectively responsed with a positive volume change to magnesium ions as an external stimulus in water. No response other than a volume change was observed in the MgCl_2_ experiment.

**Figure 7:**
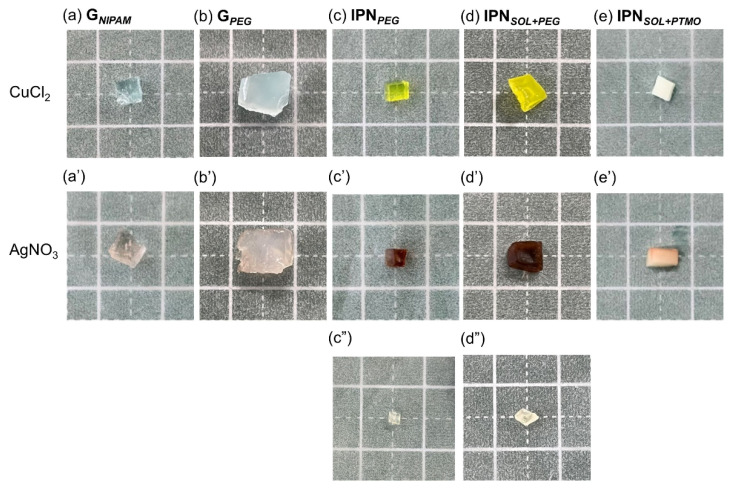
Photographs of the hydrogels: (**a**,**a’**) poly-NIPAM gel (G_NIPAM_), (**b**,**b’**) G_PEG_ prepared from DS-PEG, (**c**,**c’**) IPN_PEG_ (Table 1, run 1), (**d**,**d’**) IPN_SOL + PEG_ (run 2), and (**e**,**e’**) IPN_SOL + PTMO_ (run 4) in the presence of CuCl_2_ (upper) and AgNO_3_ (middle) (0.10 M, 24 °C) and the pristine dry gels before swelling: (**c”**) IPN_PEG_ and (**d”**) IPN_SOL + PEG_ (lower).

During the swelling experiments in aqueous solutions of transition metal salts (CuCl_2_ and AgNO_3_), the IPN hydrogels containing a PEG component underwent a unique color-change response to the metal salts as external stimuli (Figure 7), although a volume change was hardly observed.

For example, the G_NIPAM_ and G_PEG_ gels composed of poly-NIPAM and PEG networks, respectively, were isolated as pale blue hydrogels after swelling in aqueous CuCl_2_ solution (panels a and b in Figure 7). In marked contrast, the IPN_PEG_ and IPN_SOL + PEG_ IPNs, composed of poly-NIPAM and PEG components, respectively, turned yellow when immersed in aqueous CuCl_2_ solution (Figure 7c,d), whereas IPN_SOL+PTMO_ containing PTMO chains did not change color (Figure 7e). Accordingly, a color-change response to an aqueous CuCl_2_ occurred only in the gels containing poly-NIPAM and PEG. These observations suggested that a CuCl_2_ complex coordinated by both the NIPAM and the ethylene glycol units selectively formed in the interior space of the hydrogels [31,32,33,34]. Furthermore, the IPNs containing a PEG chain presented no color change when swelled in an aqueous CuSO_4_ solution.

The synthesized IPNs with a PEG component also changed color in response to AgNO_3_ as an external stimulus. When swollen in a solution of AgNO_3_, these hydrogels turned dark brown (Figure 7c’,d’), whereas the other gels (G_NIPAM_, G_PEG_, and IPN_SOL + PTMO_) scarcely changed color or afforded a pale pink hydrogel. In summary, the IPN_PEG_ and IPN_SOL + PEG_ gels showed remarkable and selective color changes in response to external stimuli of aqueous metal salts (CuCl_2_ and AgNO_3_).

We recently developed a novel multi-responsive IPN composed of poly-NIPAM and polysiloxane networks containing a chromogenic receptor for anion species. This system attained characteristic color and volume changes responding to chemical stimuli, such as acetate and/or fluoride ions, in the organic solvent *N,N*-dimethylformamide, as well as a typical temperature-responsive volume change in water [23]. The IPNs synthesized in this study, such as IPN_PEG_ and IPN_SOL + PEG_, provide another multi-responsive system to temperature and chemical stimuli of metal salts; in addition, these responsive behaviors occur in aqueous media.

## 4. Conclusions

Novel IPNs composed of poly-NIPAM and polyether (PEG or PTMO) were easily synthesized by a one-step method, where simultaneous reactions of radical gelation and condensation of alkoxysilanes attached on the polyether ends proceeded. The IPNs were formed in the presence or absence of polysiloxane-containing silanols. The obtained IPN hydrogels showed an improved mechanical property (gel strength) in comparison with a single network poly-NIPAM gel. In addition, the IPN hydrogels with a PEG chain demonstrated a synergistic effect of both networks on responsive behavior to chemical stimuli of metal salts, such as CuCl_2_ and AgNO_3_, as well as a volume change in response to temperature. The external chemical stimulus was characterized by a remarkable color change. Therefore, the IPNs having dual-responsive functionalities, reacting to temperature (depending on the VPTT of poly-NIPAM gel in water) and metal salts, were successfully developed. The simultaneous gelation system established here could contribute to facile preparation of IPN with novel structure and function, and the constructed hydrogels may be applicable to sensing materials showing a characteristic responsive behavior.

## Figures and Tables

**Table 1:** Synthesis results of the IPN gel.

Run	Polysiloxane	Polyether	Gel Abbreviation	Yield (g) ^1^	*Q* ^2^
1	none	DS-PEG	IPN_PEG_	1.12	6.2
2	poly-SOL_Ph_	DS-PEG	IPN_SOL + PEG_	0.88	6.0
3	none	DS-PTMO	IPN_PTMO_	1.15	3.9
4	poly-SOL_Ph_	DS-PTMO	IPN_SOL + PTMO_	1.10	3.3

Conditions: poly-SOL/THF = 2 mL (theoretical concentration: 1.6 M), TMPhS = 0.2 equiv./Si, DS-PEG or DS-PTMO/THF = 1.5 mL, NIPAM = 0.90 g, [NIPAM]/[BIS]/[AIBN] = 100/4/1, temperature = 60 °C, time = 24 h. ^1^ The obtained gel was immersed in THF for 2 days and in distilled water for 2 days. ^2^ In water at 30 °C.

## Data Availability

The data presented in this study are available on request from the corresponding author.
